# Antibiotic exposure prevents acquisition of beneficial metabolic functions in the preterm infant gut microbiome

**DOI:** 10.1186/s40168-022-01300-4

**Published:** 2022-07-07

**Authors:** Yanping Xu, Olivia Milburn, Traci Beiersdorfer, Lizhong Du, Henry Akinbi, David B. Haslam

**Affiliations:** 1 The Children’s Hospital, Zhejiang University School of Medicine, Hangzhou, People’s Republic of China; 2 Global Health Center, Cincinnati Children’s Hospital Medical Center (CCHMC), Cincinnati, OH USA; 3 Division of Infectious Diseases, Cincinnati Children’s Hospital Medical Center, Cincinnati, OH 45229 USA; 4 Perinatal Institute, CCHMC, Cincinnati, OH USA; 5 Department of Pediatrics, University of Cincinnati, Cincinnati, OH USA

**Keywords:** Antibiotics, Metagenomic shotgun sequencing, Microbiome, Neonate, Preterm

## Abstract

**Background:**

Aberrations in the preterm microbiome following antibiotic therapy have been reported in previous studies. The objective of this study was to probe potential underlying mechanisms between this observation and susceptibility to adverse prematurity-related outcomes.

**Results:**

Metagenomic shotgun sequencing was performed on 133 stool and 253 skin samples collected at 1 and 3 weeks of age from 68 infants born at <36 weeks postmenstrual age and birth weight <2000 g. After accounting for gestational age and maternal antibiotics, the distribution of organisms in all samples and the corresponding metabolic pathway abundance were compared between infants exposed to postnatal antibiotics and antibiotics-naïve infants.

In antibiotic-naïve infants, gestational and postnatal age imparted similar trajectories on maturation of the microbial community and associated metabolic functional capacity, with postnatal age exerting greater contribution. Antibiotic exposure was associated with reversal in maturation trajectory from the first week to the third week of age (*p*< 0.001). Butyrate-producing genera, including *Clostridium* and *Blautia*, were significantly more abundant in antibiotic-naïve neonates at 3 weeks postnatal age. Correspondingly, metabolic pathways required for short-chain fatty acid synthesis were significantly increased in antibiotic-naïve infants, but not in antibiotic-exposed neonates, at 3 weeks after birth.

**Conclusions:**

Early brief antibiotic exposure markedly disrupts developmental trajectory of the neonatal microbiome and its corresponding functional capacity. Our findings may provide a mechanistic explanation for the known associations between antibiotic use and adverse outcomes in preterm infants.

Video Abstract

**Supplementary Information:**

The online version contains supplementary material available at 10.1186/s40168-022-01300-4.

## Background

Infants’ microbiomes undergo a major transition during the perinatal period [1–3], from initial establishment through dynamic adaptations and finally a more stable microbial community profile. Although the factors that shape neonatal microbiota are still being defined, the microbiome of breast-fed vaginally delivered term infants considered optimal to support infant development [4, 5]. However, several studies have indicated that the microbiome of preterm infants differs from term infants’ and its trajectory could be more susceptible to derangement by external factors such as perinatal antibiotic exposures [6–8]. The majority of preterm births occur in settings associated with risk factors for sepsis. Consequently, most preterm infants are treated with empiric antibiotics at birth, sometimes for a prolonged period of time, because neonatal sepsis is often associated with substantial mortality and morbidity [9–13]. Deleterious impacts of perinatal exposure to antibiotics have been reported in several studies [8, 14, 15]. However, most studies that reported microbial community establishment, composition, and evolution in neonates have relied on amplicon sequencing-based (e.g., 16S rRNA) analysis on a small sample of, typically, term infants [16] and came up with differing conclusions. Furthermore, most infants in prior studies received antibiotics, making comparison to antibiotic-naïve infants problematic.

In order to increase insight into the ontogeny of the microbiome in preterm infants, especially as it relates to modifiable environmental conditions, such as antibiotic exposure, we assessed the microbiome in a prospective cohort of preterm infants, from birth till 3 weeks of age using shotgun metagenomic sequencing. We compared between three body sites (axilla, groin, and fecal material), as the organization of the microbiota in term [16] and preterm [17] infants is body site-dependent. The skin surface varies owing to regional differences in skin anatomy and, according to culture-based studies, different regions are known to support distinct sets of microorganisms. Within days after birth, rapid surface microbial colonization coincides with changes in skin barrier functions [18]. To better understand changes in microbial compositions, the dynamics of microbial interactions at skin surfaces were studied, and compared to gut microbiome. We also characterized the abundance of microbial genes that encode metabolic functions and identified metabolic pathways that are impacted by antibiotics exposure in our cohort of low gestational age neonates.

## Results

### Study population and antibiotic exposure

Samples from the skin (axilla and groin) and stool were collected from 68 preterm infants, median (range) birth weight 1384 (648–1940) g and gestational age 30.5 (24.0–35.0) weeks (Table 1). The vast majority (86.8%) of infants were exposed to maternal antibiotics, administered within 72 h prior to birth. Twenty-two (32.4%) infants received postnatal antibiotics (classified as antibiotic-exposed) while 46 (67.6%) infants did not (antibiotic-naïve; Table 1). In the first week after birth, ampicillin (18 infants, 26.5%), nafcillin (4, 5.9%), gentamicin (17, 25.0%), tobramycin (2, 2.9%), and vancomycin (2, 2.9%) were administered, while 48 infants (70.6%) received no antibiotics. In the second to the third weeks, ampicillin (1, 1.6%), nafcillin (8, 12.9%), gentamicin (1, 1.6%), tobramycin (7, 11.3%), vancomycin (2, 3.2%), and amoxicillin (1, 1.6%) were administered, while 51 infants (82.3%) received no antibiotics (Supplemental Table 1). Antibiotic-naïve and antibiotic-exposed infants were similar in demographic characteristics except for modest difference in body weights which was accounted for using generalized linear mixed modeling in subsequent analysis. Similarly, median specimen collection times were not statistically significantly different (Table 1). Most (97.1%) of the infants were fed human milk during the study period. Two neonates developed NEC in the antibiotic-exposed group during the study period. Following shotgun sequencing, and after quality control (Supplemental Table 2), 375 samples were available for downstream analysis (Supplemental Table 3—Genera, Supplemental Table 4—Species, and Supplemental Table 5—Pathways). A summary sample size by body sites, postnatal ages, gestational ages, antibiotic treatment groups, and collection times is provided in Supplemental Table 2.

**Table 1:** Demographic characteristics

	Antibiotic-naïve group (*n*=46)	Antibiotic-exposed group (*n*=22)	*p*-values
Birth weight in g, median (range)	1478.0 (688–1940)	1173.0 (648–1924)	0.003
Gestational age in weeks, median (range)	31.5 (28–35)	28.5 (24–32)	<0.001
Female gender, *n* (%)	25 (54.3)	12 (54.5)	0.988
Multiple gestations, *n* (%)	35 (76.1)	17 (77.3)	0.914
Race, *n* (%)			
White	23 (50.0)	11 (50.0)	1
Black or African American	18 (39.1)	10 (45.5)	0.62
Asian	2 (4.3)	0 (0.0)	0.321
Native Hawaiian or other Pacific Islander	1 (2.2)	0 (0.0)	0.486
Unknown or not reported	2 (4.3)	1 (4.5)	0.97
Hispanic or Latino, *n* (%)	2 (4.3)	1 (4.5)	0.97
Cesarean section, *n* (%)	42 (91.3)	20 (90.9)	0.957
Diet, *n* (%)			
Mostly breast milk	39 (84.8)	19 (86.4)	0.863
Any receipt of formula	4 (8.7)	1 (4.5)	0.54
Any receipt of breast milk	44 (95.7)	22 (100.0)	0.321
Perinatal maternal antibiotic exposure, *n* (%)	39 (84.8)	20 (90.9)	0.486
Specimen collection time in days			
Week 1	7 (5–9)	6.5 (5–9)	0.907
Week 3	16 (15–21)	17 (15–24)	0.562

### Maturation and differentiation of the microbiome

**Fig. 1:**
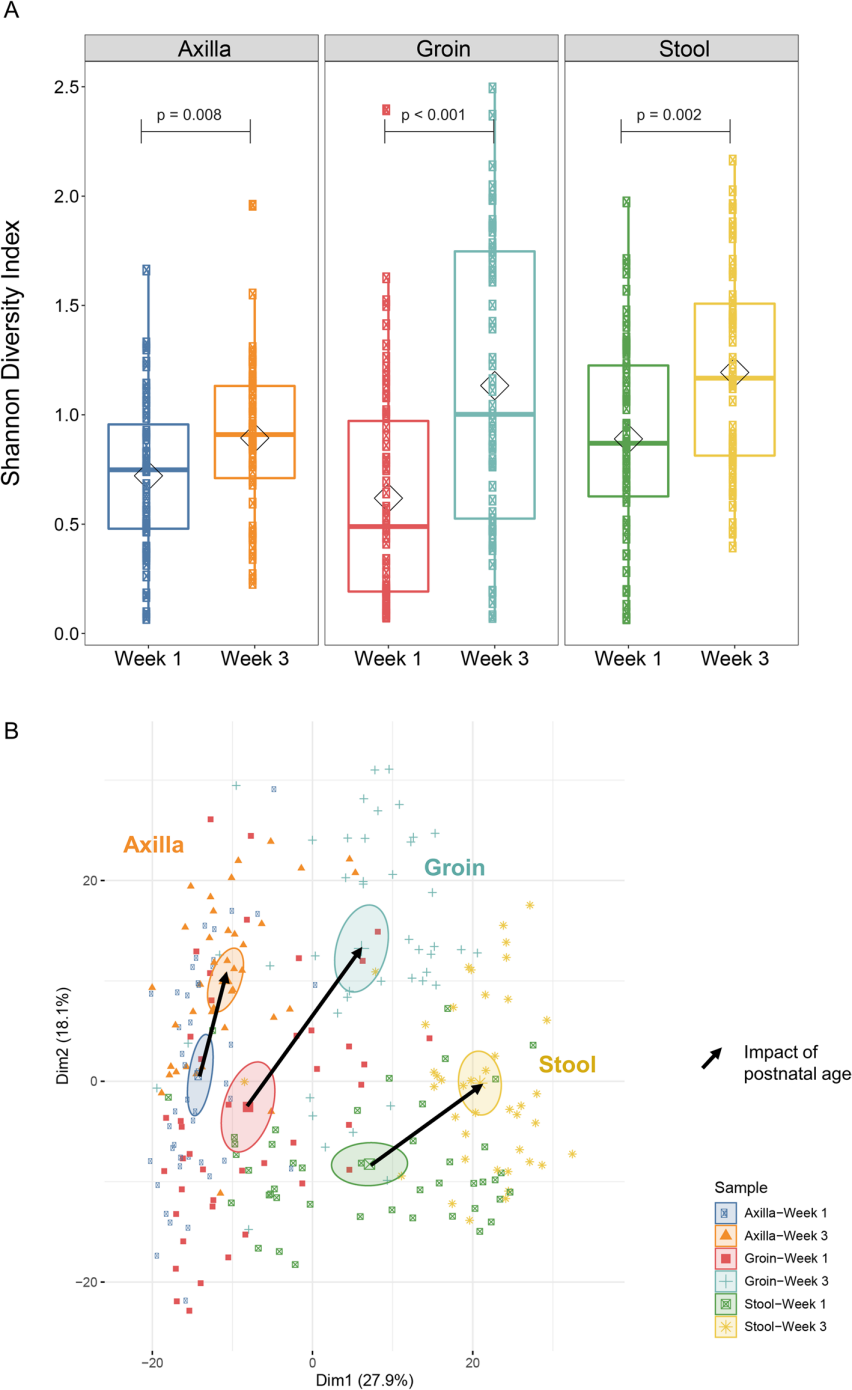
Maturation of the microbial community during the first three weeks of age. Ontogeny of overall microbiome composition was assessed in antibiotic-naïve preterm infants (*n*=46) by analyzing sequencing data for microbial diversity. **A** *Microbiome diversity*. Box and whisker plot of microbiome diversity by Shannon index using the median and interquartile ranges for each cohort. There was significantly increased microbial diversity in the week 3 compared to that in week 1; *p* = 0.008, <0.001, =0.002 in axilla (*n*=83), groin (*n*=86), and stool (*n*=89) samples respectively in antibiotic-naïve infants. **B** *Microbiome composition*. Principal component analysis (PCA) of the microbiome. Genera abundance data was subjected to generalized log2 transformation followed by PCA. Samples were then colored by group membership and ellipses represent the 95% confidence interval around the centroid of microbiome composition for each group. Arrows were drawn between the centroids to indicate the magnitude and direction of microbiome change in the first two dimensions. MRPP was used to assess the significance of difference in microbiome composition between groups. Genera composition was statistically distinct from weeks 1 to 3 between all three specimen sites, by MRPP (*P* <0.001)

To understand the ontogeny of the microbial community of the stool and skin in the preterm infant during the first 3 weeks of age, we focused on infants that did not receive postnatal antibiotics during this 3-week period. Microbial DNA from a total of 89 stool and 169 skin swabs (86 from the groin and 83 from the axillae) from 46 antibiotic-naïve infants was sequenced and data from week 1 were compared with week 3. We found, as expected, that at all three body sites examined, the number and diversity of genera increased from week 1 to week 3 (Fig. 1A). Similarly, comparison of samples at week 1 and week 3 postnatal ages by principal component analysis (PCA) revealed maturation and differentiation at the three body sites. While at week 1, samples from the three body sites were more closely clustered together, by week 3, microbial composition had become more distinct across the three sites (Fig. 1B). As might be expected, the composition of groin skin microbiome was more similar to gut microbiome than was axillary skin microbiome, especially at week 3. The composition at each body site at week 3 was distinct from composition at week 1 (multi-response permutation procedures (MRPP), *p*-values < 0.001 for week 1 versus week 3 stool, axilla, and groin microbial composition).

**Fig. 2:**
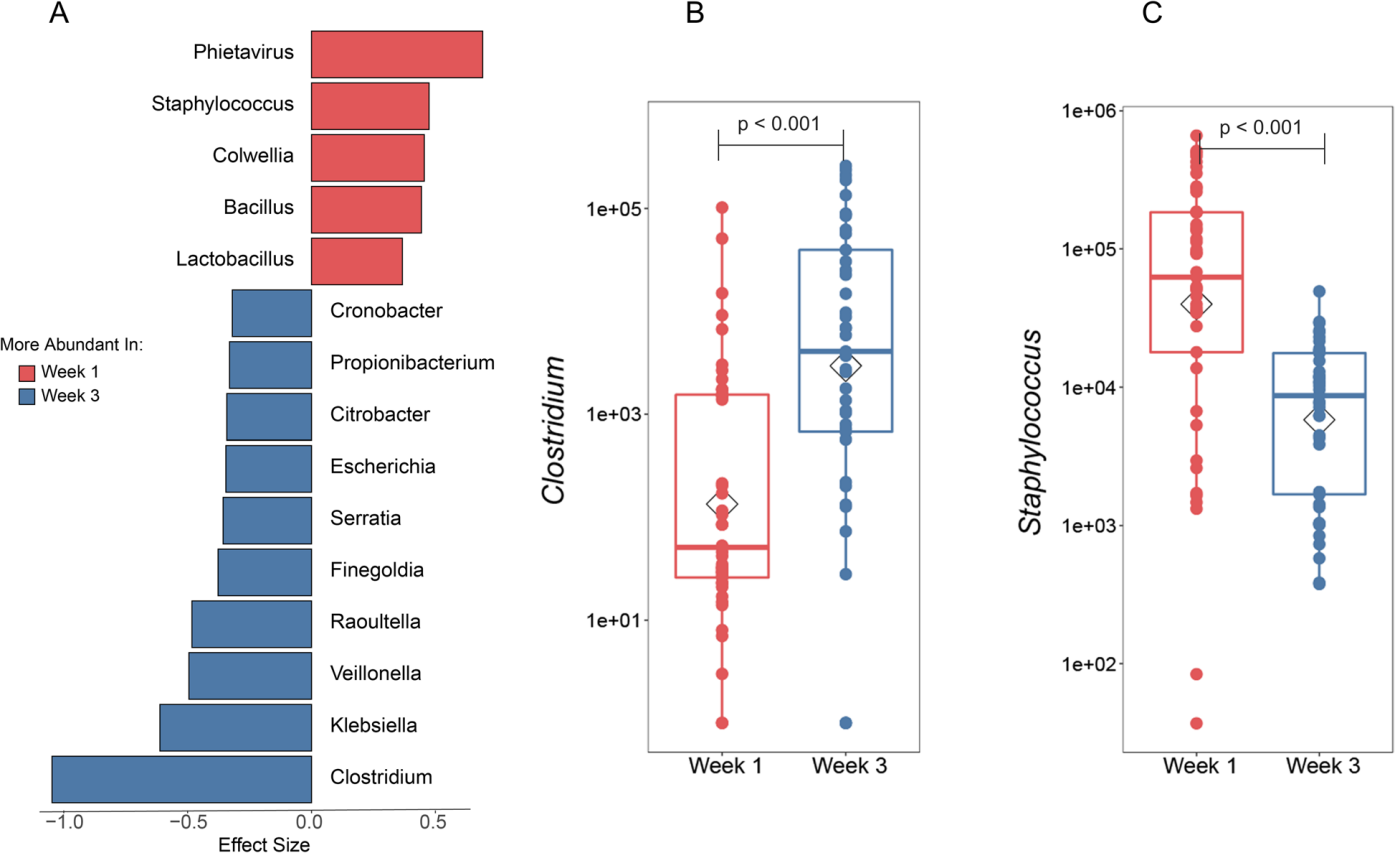
Identification of genera undergoing greatest change during postnatal maturation in antibiotic-naïve infants from week 1 to week 3. Genera that differed significantly from week 1 to week 3 in antibiotic-naïve infants were determined by pairwise Wilcoxon rank sum test followed by FDR correction for multiple testing. Among those organisms, shrinkage linear discriminant analysis was used to identify those with greatest effect size. **A** Genera that were significantly changed from week 1 to week 3 (uncorrected *p*-value < 0.05, FDR < 0.1, and effect size > 0.3) are shown for organisms more abundant at week 1 than week 3 (red) or the converse (blue). Demonstrative genera include **B** *Clostridium* and **C** *Staphylococcus*

We next investigated which organisms made the largest contribution to microbiome maturation with a focus on gut microbiome in antibiotic-naïve infants. We present organism abundance data at the genus level to reduce complexity, given the larger number of species differentially abundant between week 1 and week 3 (species-level analysis revealed 236 species that were significantly different between week 1 and week 3 with FDR < 0.05; Supplemental Table 4). Among the genera that significantly changed from week 1 to week 3, *Clostridium* demonstrated the most significance in abundance in week 3 after accounting for potential confounders (gestational age, maternal antibiotics, route of delivery, and infant diet). Several other genera, *Klebsiella*, *Veillonella*, *Serratia*, and *Escherichia*, were also significantly increased (Fig. 2A and B, all *p*<0.05). Conversely, *Staphylococcus* demonstrated the greatest decrease in abundance from week 1 to week 3 (Fig. 2C).

### Impact of gestational and postnatal ages on developmental trajectory on gut microbiome composition

We examined the contribution of gestational age to microbiome composition at week 1 and week 3 in antibiotic-naïve infants. We found diversity and overall composition of the microbiome were not significantly different between infant born at 28–32 weeks compared with 33–36 weeks’ gestational age ranges (Supplemental Figure 1A) whereas postnatal age had a significant impact on microbiome composition at all body sites among gestational age cohorts (Supplemental Figure 1B).

**Fig. 3:**
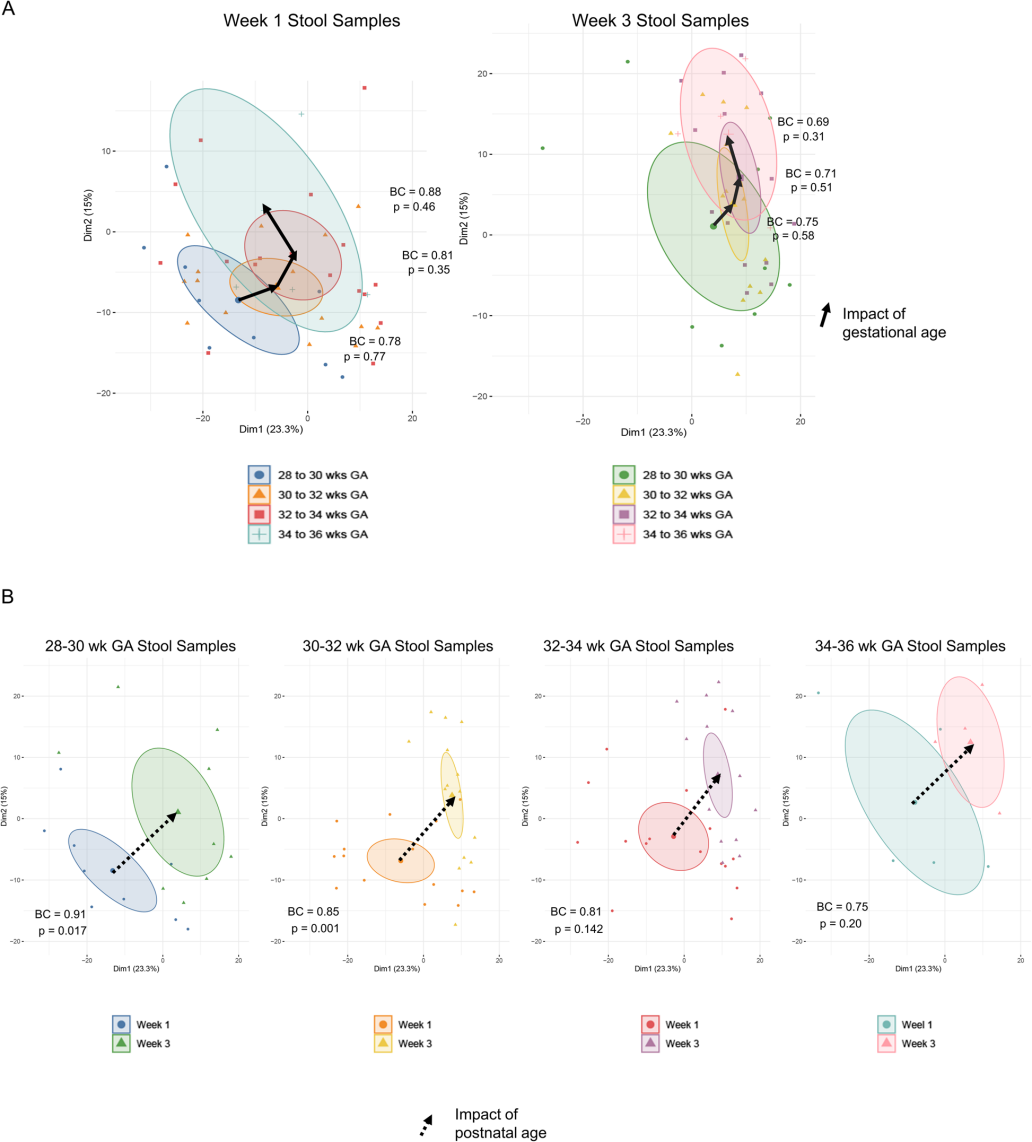
Impact of gestation and postnatal ages on developmental trajectories in antibiotic-naïve preterm infant gut microbiome. Infants were assigned to gestational age cohorts in 2-week intervals. PCA was applied to generalized log2 transformed microbiome composition data from antibiotic-naïve infants at week 1 and week 3. Samples were then colored by group membership and an ellipse was drawn at the 95% confidence interval around the group centroid. Arrows were drawn between the centroids to indicate the magnitude and direction of microbiome change in the first two dimensions. PCA calculation and graphing on the first two dimensions is the same for **A** and **B**. **A** MRPP was used to assess the significance of difference in microbiome composition between groups. Mean Bray-Curtis distance was computed for all pairwise comparisons between group members. At both week 1 and week 3, there was stepwise progression in microbiome composition across the 4 gestation age cohorts; however, none of the differences in composition between gestation cohorts attained statistical significance. Mean Bray-Curtis distances are indicated (BC). **B** Microbiome composition demonstrated progression from week 1 to week 3 in a similar trajectory in the first two dimensions at all gestational ages. Differences in composition were statistically significant at the earliest two gestational ages. Mean Bray-Curtis distances between the two groups are indicated (BC) (week 1: 28 to 30 weeks GA, *n*=25; 30^+1^ to 32 weeks GA, *n*=15; 32^+1^ to 34 weeks GA, *n*=20; 34^+1^ to 36 weeks GA, *n*=3. Week 3: 28 to 30 weeks GA, *n*=25; 30^+1^ to 32 weeks GA, *n*=15; 32^+1^ to 34 weeks GA, *n*=18; 34^+1^ to 36 weeks GA, *n*=2)

We directly compared the contribution of gestational age versus postnatal age to microbiome composition by performing unsupervised PCA, then coloring samples based on gestational age in 2-week gestational age increments, as well as postnatal age at weeks 1 and 3. Further, we calculated the mean Bray-Curtis distance of all pairwise comparisons between the 2-week gestational age cohorts. Three trends are observed in the data, although none of the differences in microbial composition between any 2-week gestational age cohorts attained statistical significance as assessed by MRPP (Fig. 3A). However, the trends for both developmental variables were in the same direction- in all cases in this PCA analysis (compare the orientation of directional arrows in Fig. 3A and B). We then compared the impact of postnatal age on microbiome composition for each gestational age (GA) cohort in antibiotic-naïve infants. Microbiome composition demonstrated progression from week 1 to week 3 at all gestational ages (Fig. 3B). Postnatal age-dependent differences in microbiome composition were greatest at the earliest gestational ages (*p*=0.017 for 28-to-30-week GA infants and *p*=0.001 for 30-to-32-week infants) and declined with advancing gestational age (Fig. 3B). Overall, the data indicate that postnatal age had a greater impact on microbiome composition than gestational age, but there is a trend toward increased contribution of gestational age later in pregnancy. We also compared the contribution of gender to microbiome composition by performing unsupervised PCA. We found the only time point with a significant difference, based on gender, was the groin at week 1 (*p*=0.002). The other body sites and time points were not significantly different with respect to gender (Supplemental Figure 2).

### Effect of antibiotic treatment on microbiome diversification and maturation

**Fig. 4:**
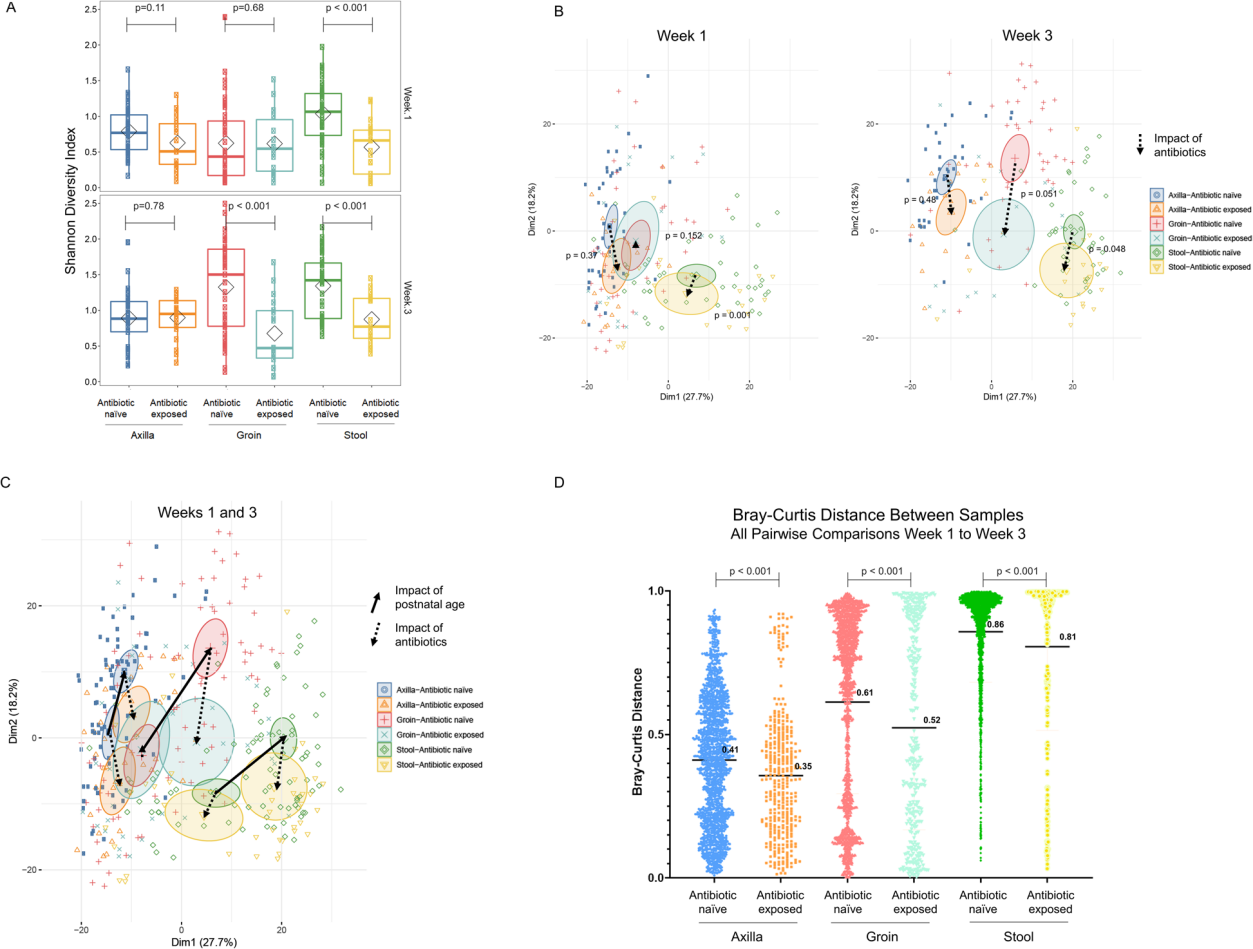
Impact of postnatal antibiotic exposure on skin and gut microbiome diversity and maturation. **A** *Microbiuome diversity*. Box and whisker plot of microbiome diversity by Shannon Index using the median and interquartile ranges for each cohort. Stool samples showed a decrease in diversity during the first and third weeks after antibiotic therapy, while skin samples from the groin demonstrated decreased diversity by the third week post-antibiotic use (*p*<0.001, week 1: axilla (*n*=43), groin (*n*=43), and stool (*n*=46) samples in antibiotic-naïve infants *vs* axilla (*n*=21), groin (*n*=21), and stool (*n*=22) samples in antibiotic-exposed infants; week 3: axilla (*n*=40), groin (*n*=43), and stool (*n*=43) samples in antibiotic-naïve infants *vs* axilla (*n*=21), groin (*n*=21), and stool (*n*=22) samples in antibiotic-exposed infants). **B** *Microbiome composition*. PCA was applied to generalized log2 transformed microbiome composition data from antibiotic-naïve and exposed infants at week 1 and week 3. Samples were then colored by group membership and an ellipse was drawn at the 95% confidence interval around the group centroid. PCA calculation and graphing in the first two dimensions is the same for **A** and **B**. Dotted arrows were drawn between the centroids to indicate the magnitude and direction of microbiome difference in the first two dimensions between antibiotic-exposed and antibiotic-naïve infants. MRPP was used to assess the significance of difference in microbiome composition between groups. **A** At week 1, the composition of gut microbiome was significantly different among antibiotic-exposed infants when compared to antibiotic-naïve infants. At week 3, the composition of groin and gut microbiome was significantly different in antibiotic-exposed and antibiotic-naïve infants. **C** Overlay of week 1 and week 3 data reveals that antibiotic exposure (dotted arrows) tends to drive microbiome composition in a direction opposite or perpendicular to postnatal age (solid arrows), suggesting that antibiotics blunt the impact of postnatal age on microbiome composition. **D** Mean Bray-Curtis distances for all pairwise comparisons from week 1 to week 3 were determined in samples from the axilla, groin, and stool in antibiotic-naïve and antibiotic-exposed infants. The mean Bray-Curtis distance is indicated as a solid line. There was a statistically significant decrease in mean Bray-Curtis distance at each body site in antibiotic-exposed infants compared to antibiotic-naïve infants (unpaired *t*-test *p* < 0.05) indicating that antibiotic exposure blunts microbiome differentiation from week 1 to week 3 at all three body sites

We next sought to understand how postnatal antibiotic exposure impacted composition and maturation of the preterm infant gut and skin microbiota. Unlike prior investigations of the preterm infant microbiome, the majority of infants in our study received no antibiotics up to 3 weeks of age. Among those that received postnatal antibiotics, the duration and intensity were generally short. Indeed, among the 22 infants that received antibiotics, 16 infants (72.7%) received < 48 h of ampicillin and gentamicin, and predominantly during the first 3 days of age. We first compared microbiome diversity across body sites at week 1 and week 3 between antibiotic-exposed and antibiotic-naïve groups. Stool samples showed a decrease in diversity during the first and third weeks and skin samples from the groin demonstrated decreased diversity by the third week post-antibiotic therapy (*p*<0.001, Fig. 4A). There were no significant differences in diversity of microbiota from the axillae at week 1 and week 3 between antibiotic-exposed and antibiotic-naïve groups. We next examined overall microbial composition using PCA. There was decreased differentiation of the gut microbial composition in antibiotic-exposed infants compared to antibiotic-naïve infants (Fig. 4B). Overlay of the PCA plots from week 1 and week 3 demonstrates that antibiotic treatment tends to shift microbial composition in the opposite direction relative to changes seen with postnatal age, indicating that antibiotics therapy is associated with blunting of the maturation of the microbiome at all three body sites (Fig. 4C). In addition, microbiome maturation across body sites measured by Bray–Curtis distance shows significant decrease in differentiation (*p* < 0.001) between antibiotic-exposed and antibiotic-naïve groups from week 1 to week 3 (Fig. 4D).

**Fig. 5:**
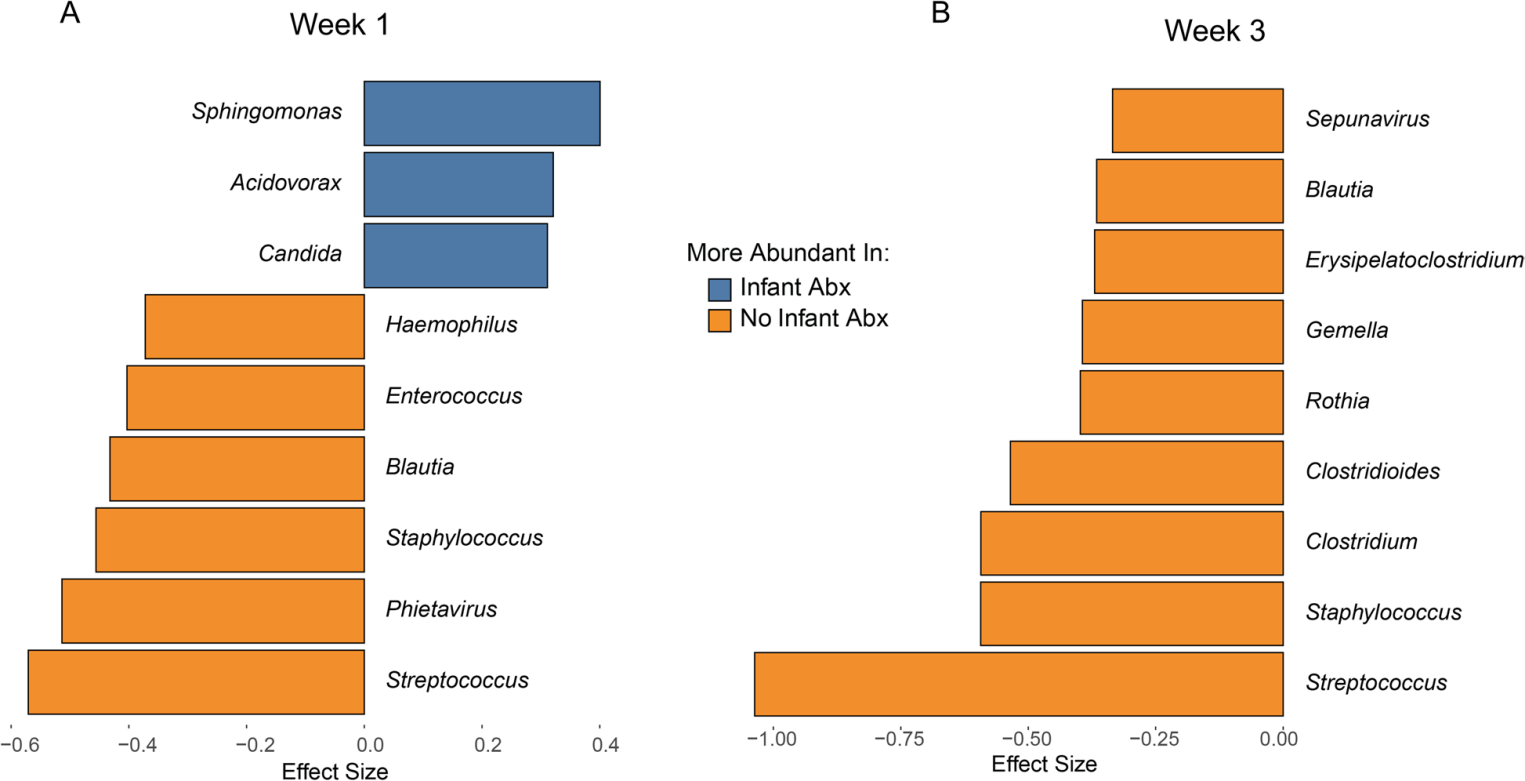
Identification of genera most impacted by antibiotic exposure in the preterm infant gut microbiome at week 1 and week 3. Genera that significantly differed in antibiotic-naïve and antibiotic-exposed infants at week 1 to week 3 was determined by pairwise Wilcoxon rank sum test followed by FDR correction for multiple testing. Among those organisms, shrinkage linear discriminant analysis was used to identify those with greatest effect size. Genera that were significantly differed in antibiotic-naïve and antibiotic-exposed infants (uncorrected *p*-value < 0.05, FDR < 0.1, and effect size > 0.3) are shown for organisms more abundant in antibiotic-exposed infants 3 (blue) or antibiotic-naïve infants (gold)

**Fig. 6:**
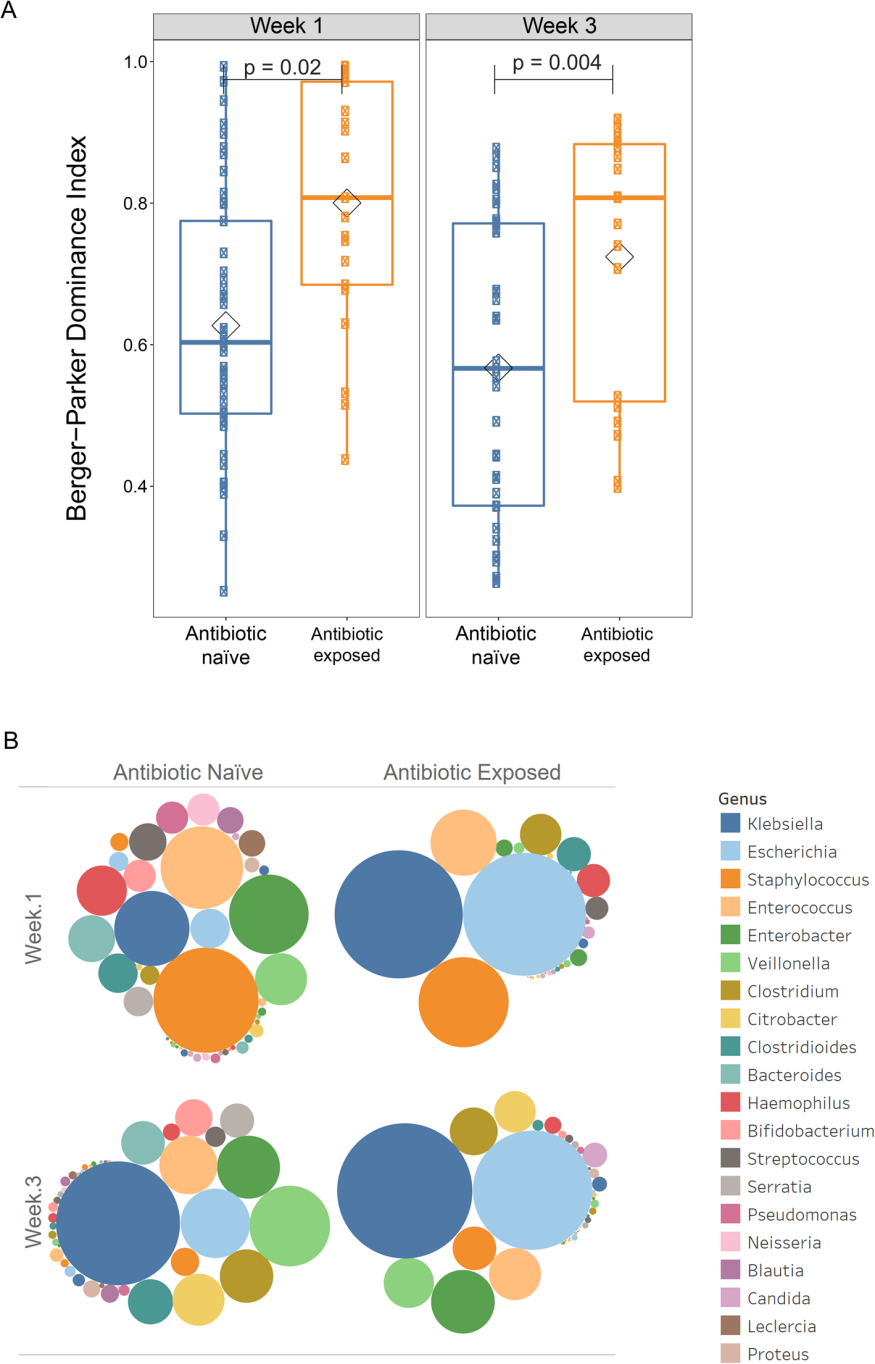
Association of antibiotic exposure with intestinal dominance by *E. coli* and *Klebsiella*. **A** Berger-Parker dominance index between the antibiotic-naïve and antibiotic-exposed infants both at week 1 and week 3 (*p*-value=0.02 and 0.004). **B** Bubble chart demonstrating the composition of preterm infant gut microbiome at weeks 1 and 3 in antibiotic-naïve and antibiotic-exposed infants

To identify organisms most impacted by antibiotic exposure, we performed ZINB-GLMM with FDR correction followed by calculation of effect size using SLDA to identify genera that differed between the microbial composition of antibiotic-exposed versus antibiotic-naïve infants at weeks 1 and 3, after accounting for gestational age, maternal antibiotics, breast milk receipt, and delivery mode. We found in gut microbiota samples that *Sphingomonas*, *Acidovorax*, and *Candida* were significantly enriched in the antibiotic-exposed group, whereas several genera, including *Blautia*, *Streptococcus*, *Enterococcus*, and *Staphylococcus*, were significantly more abundant in the antibiotic-naïve infants in the first week after birth (Fig. 5A and Supplemental Figure 3). At week 3, no genus was significantly higher in abundance in antibiotic-exposed infants, while several genera including *Clostridium*, *Clostridioides*, *Blautia*, *Streptococcus*, and *Staphylococcus* were significantly increased in the antibiotic-naïve infants at 3 weeks after birth in stool samples (Fig. 5B and Supplemental Figure 3). Antibiotic exposure resulted in domination of the gut microbiome by a small number of genera, as indicated by the Berger-Parker Dominance index both at week 1 and week 3 (Fig. 6A). In antibiotic-treated infants, *Escherichia coli* and *Klebsiella* dominate at both time points (Fig. 6B).

### Effect of antibiotic exposure on maturation of metabolic functional capacity of gut microbiome

**Fig. 7:**
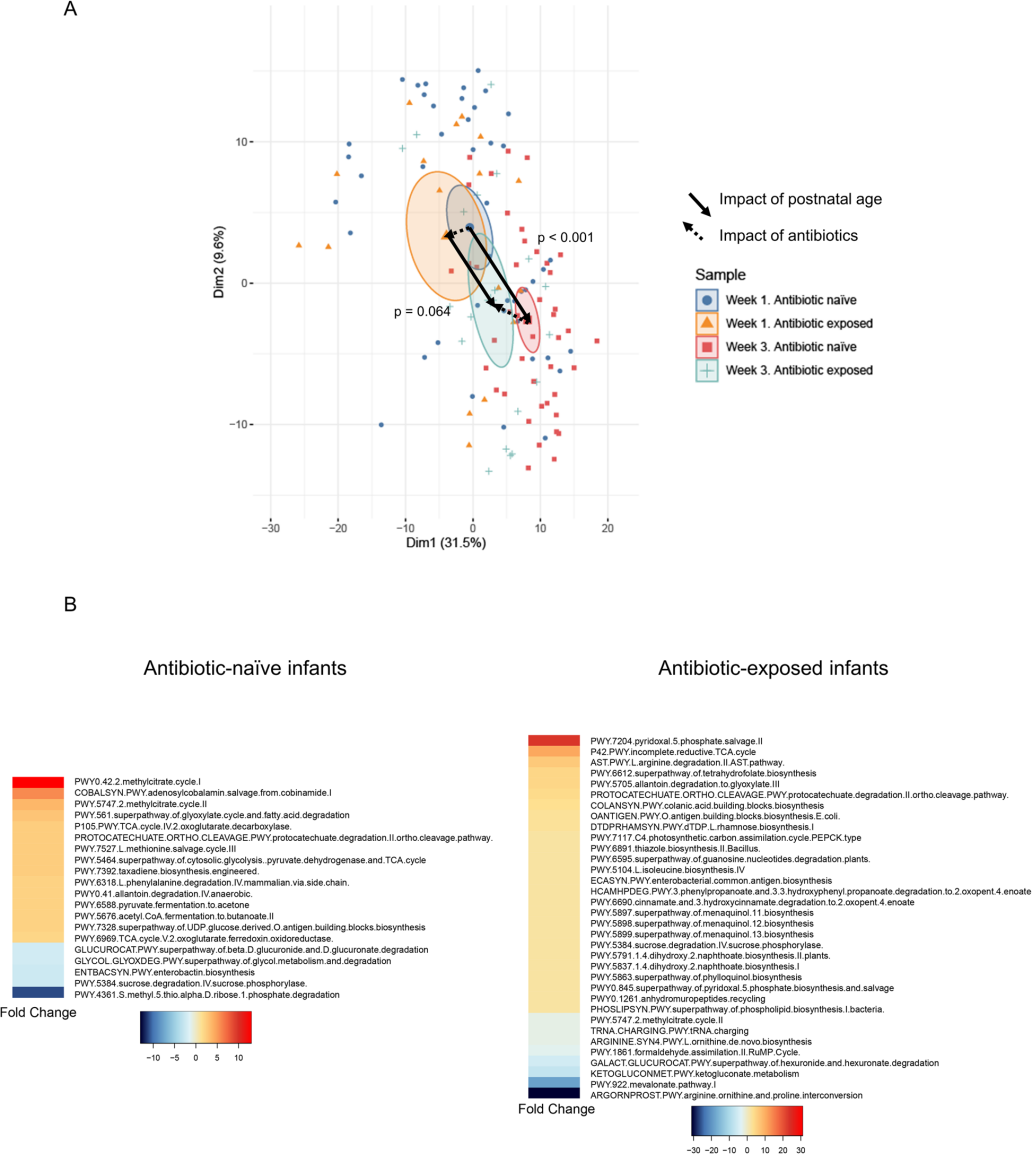
Maturation of metabolic functional capacity of gut microbiome as function of postnatal age and antibiotic exposure. **A** Principal component analysis (PCA) of metabolic pathway composition. Overall composition of metabolic pathway genes and their maturation over 3 weeks was assessed in stools from antibiotic-naïve preterm infants and compared to antibiotic-exposed assessed by unsupervised PCA**.** Normalized pathway abundance data were subjected to generalized log2 transformation then PCA. Samples were then colored by group membership and an ellipse was drawn at the 95% confidence interval around the group centroid. Arrows were drawn between the centroids to indicate the magnitude and direction of microbiome change in the first two dimensions. MRPP was used to determine significance of the difference in pathway abundance between groups. A. There was a statistically significant difference in metabolic functional capacity at week 1 compared to week 3 in antibiotic-naïve infants (*p* < 0.001). In contrast, there was a smaller and non-significant change in metabolic pathway composition in antibiotic-exposed infants from week 1 to week 3 (*p*= 0.064). **B** Heatmap demonstrating the fold change of metabolic pathways that differ from week 1 to week 3 in antibiotic-naïve and exposed infants. Generalized linear mixed modeling with fixed and random effects (GLMM) was used to identify metabolic pathways that significantly differed from week 1 to week 3 in antibiotic-naïve and exposed infants. After accounting for maternal antibiotics, gestational age, delivery mode, and receipt of breast milk, genes encoding a total of 20 metabolic pathways were significantly different and had fold-change > 2 from week 1 to week 3 in antibiotic-naïve infants. In contrast, 34 metabolic pathways genes were significantly different in infants that received antibiotics

**Fig. 8:**
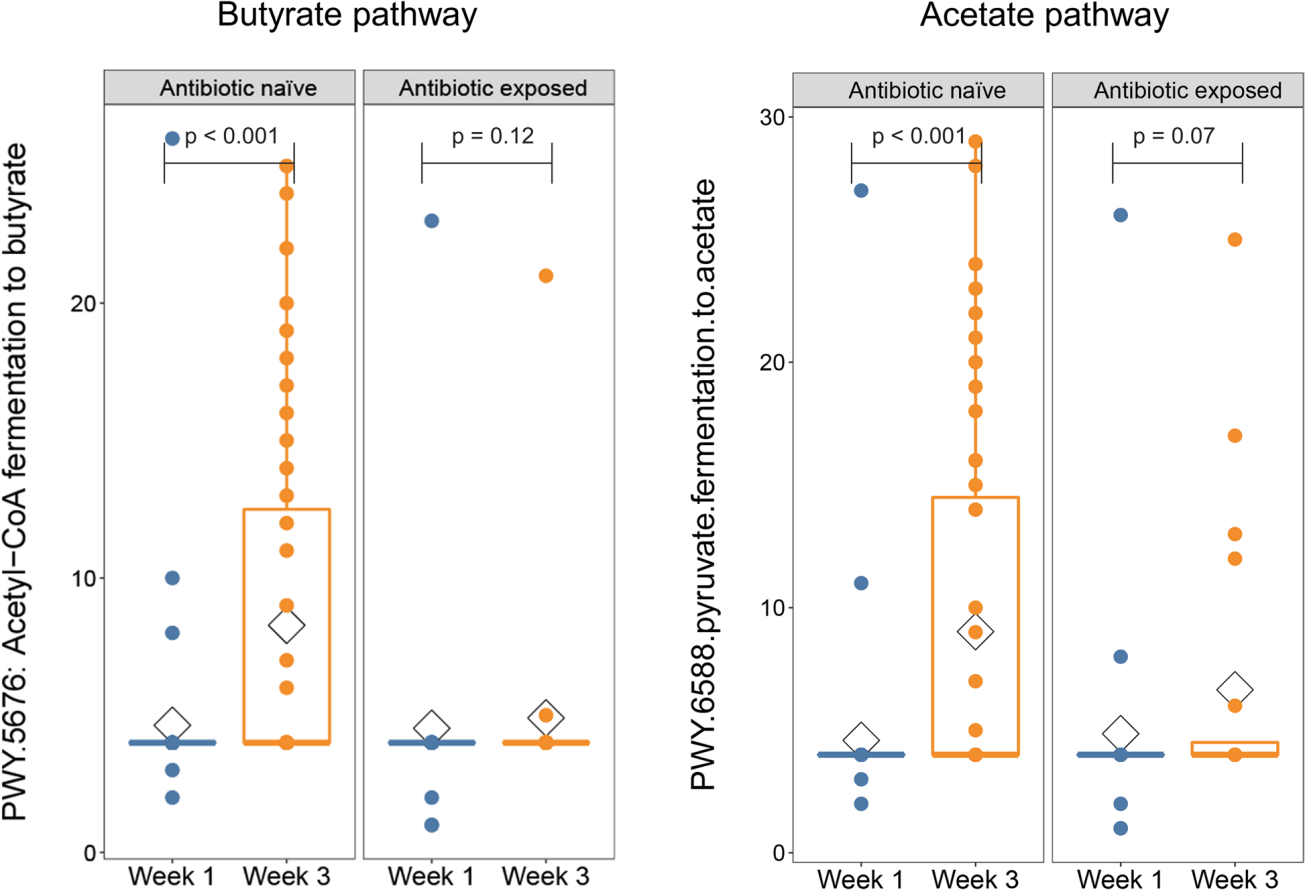
Acquisition of a major butyrate and acetate synthetic pathway in the preterm infant gut microbiome was markedly impaired in antibiotic-exposed infants. The relative abundance of metabolic pathway genes was compared from week 1 to week 3 in antibiotic-exposed and antibiotic-naïve infants using GLMM. The acetyl-CoA to butyrate and acetate synthesis pathway, a contributor to butyrate and acetate synthesis in meconium stools from infants, was significantly increased in abundance from week 1 to week 3 in antibiotic-naïve infants. Acquisition of the same pathway was markedly blunted in antibiotic-exposed infants

Microbes are believed to influence human health, in part, through their ability to produce metabolites with either beneficial or harmful effects on the host. Given the primacy of metabolic activity in the preterm infant gut microbiota and potential direct impact on health outcome, we characterized the metabolic pathways content in antibiotic-naïve versus antibiotic-exposed. DNA reads were assigned to metabolic pathways using HUMAnN2 [19]. To assess overall metabolic pathway abundance, we used unsupervised PCA comparing samples based on the infant’s postnatal age and antibiotic exposure status (Fig. 7A). There was a significant change in the distribution of metabolic pathways represented in the preterm infant gut between week 1 and week 3 in antibiotic-naïve infants (*p*<0.001), indicating the metabolic capacity demonstrated significant functional maturation in these infants. However, the change from week 1 to week 3 in antibiotic-exposed infants was not significant (*p*=0.064), indicating that antibiotics impaired maturation of metabolic functional capacity. On PCA analysis, antibiotic exposure was associated with a shift to a more immature metabolic pathway abundance at week 3 compared to antibiotic-naïve infants. These two features of the PCA plot reveal that antibiotic exposure is associated with stunting of functional maturation of the preterm infant microbiome. To specifically address the role of antibiotic exposure and account for differences in gestational age and maternal antibiotic exposure between groups, we employed generalized linear mixed modeling (GLMM) with fixed and random effects. We found that genes encoding a total of 20 metabolic pathways were significantly different from week 1 to week 3 in the stools of antibiotic-naïve infants. In contrast, 34 metabolic pathways were significantly different in infants that received antibiotics (Fig. 7B). Notably, there was very little overlap in the metabolic pathways enriched from week 1 to week 3 in antibiotic-naïve versus antibiotic-exposed infants (Supplemental Figure 4) indicating that antibiotic exposure is associated with profoundly different trajectories in the development of metabolic functional pathways in the preterm infant gut. Among metabolic pathway genes that were significantly increased in antibiotic-naïve infants were pathways involved in synthesis of the short-chain fatty acids butyrate (*PWY.5676. acetyl.CoA.fermentation.to.butanoate.II*) and acetate (*PWY.6588.pyruvate.fermentation.to.acetate*) (Fig. 8 and Supplemental Table 5). It is particularly notable that antibiotic exposure completely suppressed increased abundance of the butyrate and acetate synthesis pathways seen in antibiotic-naïve infants, suggesting that maturation of the gut microbiota from week 1 to week 3 is associated with increased capacity to produce metabolites beneficial to the developing neonatal intestinal epithelium (Fig. 8). HUMAnN2 was unable to unambiguously identify organisms that contributed the most to short-chain fatty acid synthesis; however, *Clostridium* and *Blautia* species are major short-chain fatty acid producers in the healthy gut microbiome [20, 21] and both were increased markedly in abundance in the gut of antibiotic-naïve infants from week 1 to week 3 (Fig. 2A). Failure of this maturation in the gut microbiome of antibiotic-exposed infants (Fig. 5B and Supplemental Figure 3) likely accounts for the marked defect in short-chain fatty acid synthesis capacity in antibiotic-exposed infants.

## Discussion

Antibiotics are the most-commonly prescribed medications in Neonatal Intensive Care Units. Previous reports have described the effects of antibiotics on the infant microbiome maturation but implication on microbial functional capacity has not been adequately studied in preterm infants. In this prospective population study, in addition to altered maturation and differentiation, the functional capacity of the micobiome was deranged by short-term exposure of preterm infants to antibiotics in the immediate postnatal period. Postnatal age plays a greater role than gestational age in shaping the microbiota in the gut and skin. Acquisition of putatively beneficial metabolic pathways was particularly impacted following brief antibiotic exposure, as exemplified by blunting of genes associated with butyrate and acetate synthesis at week 3 in the microbiota of antibiotic-exposed infants. Alteration in the trajectory of microbial metabolic pathways may contribute to the pathomechanism of antibiotic-associated prematurity-related adverse events, such as NEC and late-onset sepsis [22]. These data also add to the growing body of evidence of the potential deleterious effects of antibiotic therapy on the abundance and diversity of the developing microbiome of premature infants.

The developmental trajectory to microbiome maturation is probably mainly innate, but it is influenced by the physiologic state of the developing intestine and the environment. Exposure to antibiotics appears to alter the developmental program. In agreement with previous reports [23–27] and contrary to others [28–30], gestational age at birth appeared to exert less influence on the microbiota development than postnatal age. In our study, both gestational age and postnatal age independently exerted effects on the preterm infant microbiome. However, postnatal age had a greater impact on microbiome maturation than gestational age. There were differences in the microbiome in skin samples from the groin between the genders in week 1. The reason for these differences is not clear. While we may speculate that anatomic differences could play some role, it is unclear why those differences would disappear by week 3. Similar sex-specific differences in microbial pathways were also reported in a small sample of healthy preterm infants [31]. Our cohort comprises very low birth weight infants and we specifically assessed the role of brief early antibiotic exposure on the microbiome and its metabolic profiles. It is difficult to compare our findings with the few studies in the literature.

A major link between microbiota and host cells is the production of short-chain fatty acids through bacterial metabolism [32–34]. We demonstrated that at week 3 several genera that are beneficial to the host, including *Blautia* and *Clostridium*, were significantly increased in antibiotic-naïve infants. *Blautia* is a dominant genus in the human gut microbiota and belongs to the clostridial subcluster XIVa. Along with *Clostridium*, *Blautia* is a main butyrate-producing bacterium that is associated with the host’s metabolic function [20, 21]. Butyrate plays a pleiotropic role in the gut, including enhancing epithelial barrier integrity, and inhibition of inflammation of colonocytes. Alterations in *Blautia* and *Clostridium* abundance may also be important in the pathogenesis of Crohn’s disease, which, similar to NEC, is associated with disrupted intestinal barrier function [35]. In our study, we demonstrate that butyrate and acetate synthesis genes are enriched at the critical week 3 timepoint, while this enrichment is completely abrogated in antibiotic-exposed infants. Taken together, our findings have implications for a putative host-microbe crosstalk pathway wherein protective butyrate signaling is dampened by antibiotic therapy.

Despite the relatively large sample size of our cohort, this is a single institution study. The vast majority of our infants were exposed to perinatal maternal antibiotics administered within 72 h of delivery and most of the infants were delivered by cesarean section. These may limit the generalizability of our findings. However, most VLBW infants are products of high-risk pregnancies, which are often associated with high rate of cesarean births. On the upside, most infants in our cohort were not treated with antibiotics postnatally. This enabled us to characterize the ontogeny of the microbiome in preterm infants and to assess the impact of gestational and postnatal age on microbiome composition and metabolic functional capacity. Unlike previous studies that relied on 16S RNA sequencing, we used high resolution shotgun metagenomic sequencing and multidimensional analysis platforms to assess microbial species composition and the corresponding metabolic functional genes.

## Conclusions

Our data demonstrate that brief early antibiotic therapy to preterm neonates engenders alterations in microbial composition and differentiation with resulting in delay in acquisition of beneficial microbial metabolic pathways.

## Methods

### Study subjects and sample collection

The study was approved by the Institutional Review Boards of Cincinnati Children’s Hospital Medical Center and the University of Cincinnati (CCHMC #2018-7474). Infants born at <36 weeks postmenstrual age and birth weight <2000 g admitted to the Neonatal Intensive Care Unit (NICU) at the University of Cincinnati Medical Center (UCMC) from February 2019 to October 2019, with anticipated length of stay of >21 days, were enrolled on admission. Infants were stratified into (1) antibiotic-exposed or (2) antibiotic-naïve groups, based on any postnatal antibiotic therapy. Stool and skin samples were collected at 1 (5–9 days) and 3 (15–21 days) weeks of postnatal age following written consent from parents. Skin samples were obtained with a soft cotton swab moistened in normal saline from infants’ groins and axillae. Swab tips were snapped off into sterile 1.5-ml polyethylene tubes, transferred immediately to a −80°C freezer for storage. Stool samples were scrapped from diapers and immediately frozen at −80°C until analysis. Maternal and neonatal demographic data, labor complications, and NICU courses, including postnatal antibiotic therapy, were extracted from electronic medical records.

### Illumina sequencing

Briefly, genomic DNA was isolated from stool samples using the Qiagen QIAamp® PowerFecal® Kit, per the manufacturer’s protocol. For skin samples, MasterPure yeast DNA purification kit (Lucigen Corp. Middleton, WI) and Genomic DNA Mini Kit (Invitrogen, Carlsbad, CA), lysis buffer with the addition of 1ul (3500 U) of diluted Ready Lyse solution were used according to the manufacturer’s protocol [36]. Yield and purity of isolated DNA were confirmed by Qubit analyzer (Invitrogen, Carlsbad, CA) [37]. Amplified library generation was performed with Nextera XT adapters, and sequencing performed to obtain 150-bp DNA paired end reads to a depth of approximately 2 million reads per sample using Illumina NextSeq 500 (Illumina, Inc., San Diego, CA) in the Microbial Genomics and Metagenomics Core at Cincinnati Children’s Hospital.

### Data analysis

Raw sequence reads were extracted and demultiplexed using the Illumina program bcl2fastq. Raw reads were then filtered and trimmed for quality control using the program Sickle with default settings [38]. Trimmed reads were aligned using Kraken [25] to a custom microbial genome database (that includes all RefSeq bacterial, fungal, parasitic, and viral genomes supplemented with additional bacterial and fungal genome sequences from the National Center for Bioinformatics to determine quantitative genus and species abundance for more than 40,000 microbial species genomes). An exact sequence read match of k-mer length 32 was used in Kraken2 to assign reads to the lowest common ancestor. Samples with less than 100,000 assigned reads were excluded from further analysis. Normalization of count data was performed using rrarefy with the Vegan package in R to give the normalized abundance at both the genus and species level [39]. Thereafter, species or genera that were absent in more than 95% of the samples or contributed less than 1% to the overall microbial abundance were excluded. To establish differences in gut and skin microbiota between antibiotic-exposed versus antibiotic-naïve groups and between time points, shotgun metagenome sequence data were analyzed using multi-response permutation procedures (MRPP) [40]. Visualization of overall microbiome composition among groups was performed using PCA. Examination of clinical covariates was performed using zero inflated negative binomial generalized linear mixed models (ZINB-GLMM). Generalized linear mixed modeling was performed using the “glmer” command from the lme4 package in R. To identify individual genera that differ between groups, we performed non-parametric tests (Mann-Whitney *U* test or Kruskal-Wallis test), followed by the Bonferroni correction for multiple testing. Effect size of the statistically significantly different genera was determined using shrinkage linear discriminant analysis (SLDA). We performed generalized linear mixed modeling with parameterization for (NBZI-GLMM), then performed false discovery rate (FDR) correction followed by calculation of effect size using SLDA as we described previously [41]. All data analyses were carried out using the R programming language or SPSS software for Windows (version 20.0; SPSS, Inc., Chicago, IL, USA).

## Supplementary Information

**Additional file 1: Table S1**. Detailed information on all infant antibiotic exposures.

**Additional file 2: Table S2**. Summary sample size by body sites, postnatal ages, gestational ages, antibiotic treatment groups, and collection times.

**Additional file 3: Table S3**. Raw genus count data.

**Additional file 4: Table S4**. Raw species count data.

**Additional file 5: Table S5**. Microbial pathway abundance data.

**Additional file 6: Figure S1**. A Impact of gestational age on microbiome structure, using PCA. B Impact of postnatal age on microbiome structure. **Figure S2**. The contribution of gender to microbiome composition at all body sites and found the only time point with difference in gender was groin at Week 1 (*p*=0.002, other *p*>0.05). **Figure S3**. Antibiotic exposure was associated with altered abundance of several genera. **Figure S4**. Microbial metabolic pathway abundance and enrichment from Week 1 to Week 3 were compared between antibiotic-exposed and antibiotic-naïve preterm infants using MRPP and GLMM, as described in the text.

## Data Availability

Sequence data have been deposited to the NCBI Sequence Read Archive and are available under accession number SUB10638802. Linux shell and R programming language scripts used to analyze the data and generate the manuscript figures are available upon request.
