# Supplementary material for: Antibiotic exposure prevents acquisition of beneficial metabolic functions in the preterm infant gut microbiome
Source: Microbiome. 2022 Jul 7;10:103. doi: 10.1186/s40168-022-01300-4 (PMC9260971; doi:10.1186/s40168-022-01300-4)
Supplement: Supplementary file 7 — Additional file 6: Figure S1. A Impact of gestational age on microbiome structure, using PCA. B Impact of postnatal age on microbiome structure. Figure S2. The contribution of gender to microbiome composition at all body sites and found the only time point with difference in gender was groin at Week 1 (p=0.002, other p>0.05). Figure S3. Antibiotic exposure was associated with altered abundance of several genera. Figure S4. Microbial metabolic pathway abundance and enrichment from Week 1 to Week 3 were compared between antibiotic-exposed and antibiotic-naïve preterm infants using MRPP and GLMM, as described in the text. [file 40168_2022_1300_MOESM6_ESM.docx]

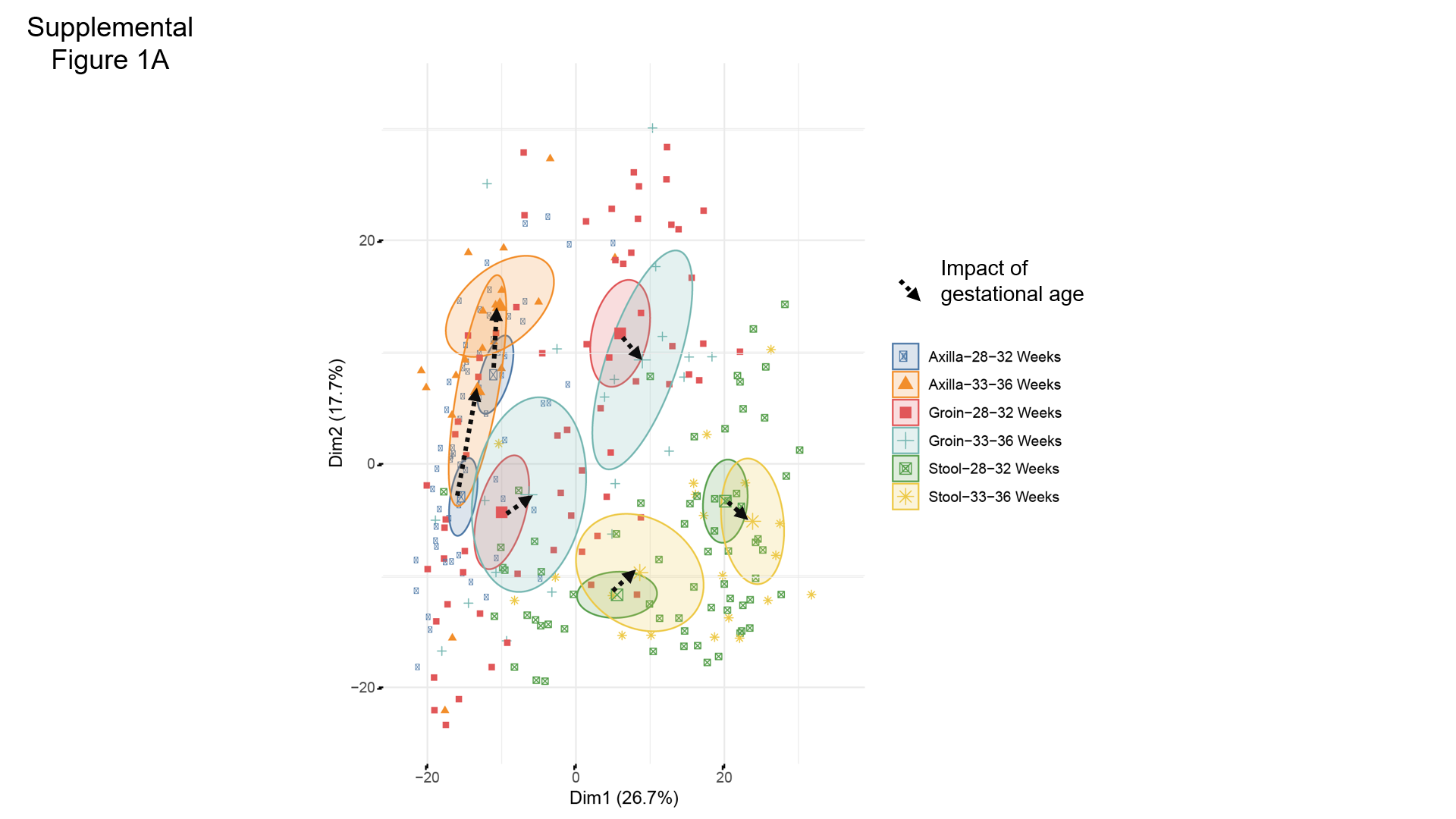


Supplemental Figure 1. Impact of gestational age on microbiome structure, using PCA. PCA was applied to generalized log2 transformed microbiome composition data from antibiotic-naïve and exposed infants at week 1 and week 3. Samples were then colored by group membership and an ellipse was drawn at the 95% confidence interval around the group centroid. PCA calculation and graphing in the first two dimensions is the same for Supplemental Figures 1A and 1B. Dotted arrows were drawn between the centroids to indicate the magnitude and direction of microbiome difference in the first two dimensions between infants in young and older gestational age cohorts while solid lines indicate the magnitude and direction of differences in microbiome composition from week 1 to week 3. MRPP was used to assess the significance of difference in microbiome composition between groups. A. Diversity and overall composition were not significantly different between the cohorts at any body site. (Axilla: 28 to 32 wks GA, n=65; Axilla: 33 to 36 wks GA, n=18; Groin: 28 to 32 wks GA, n=66; Groin: 33 to 36 wks GA, n=20; Stool: 28 to 32 wks GA, n=68; Stool: 33 to 36 wks GA, n=21).


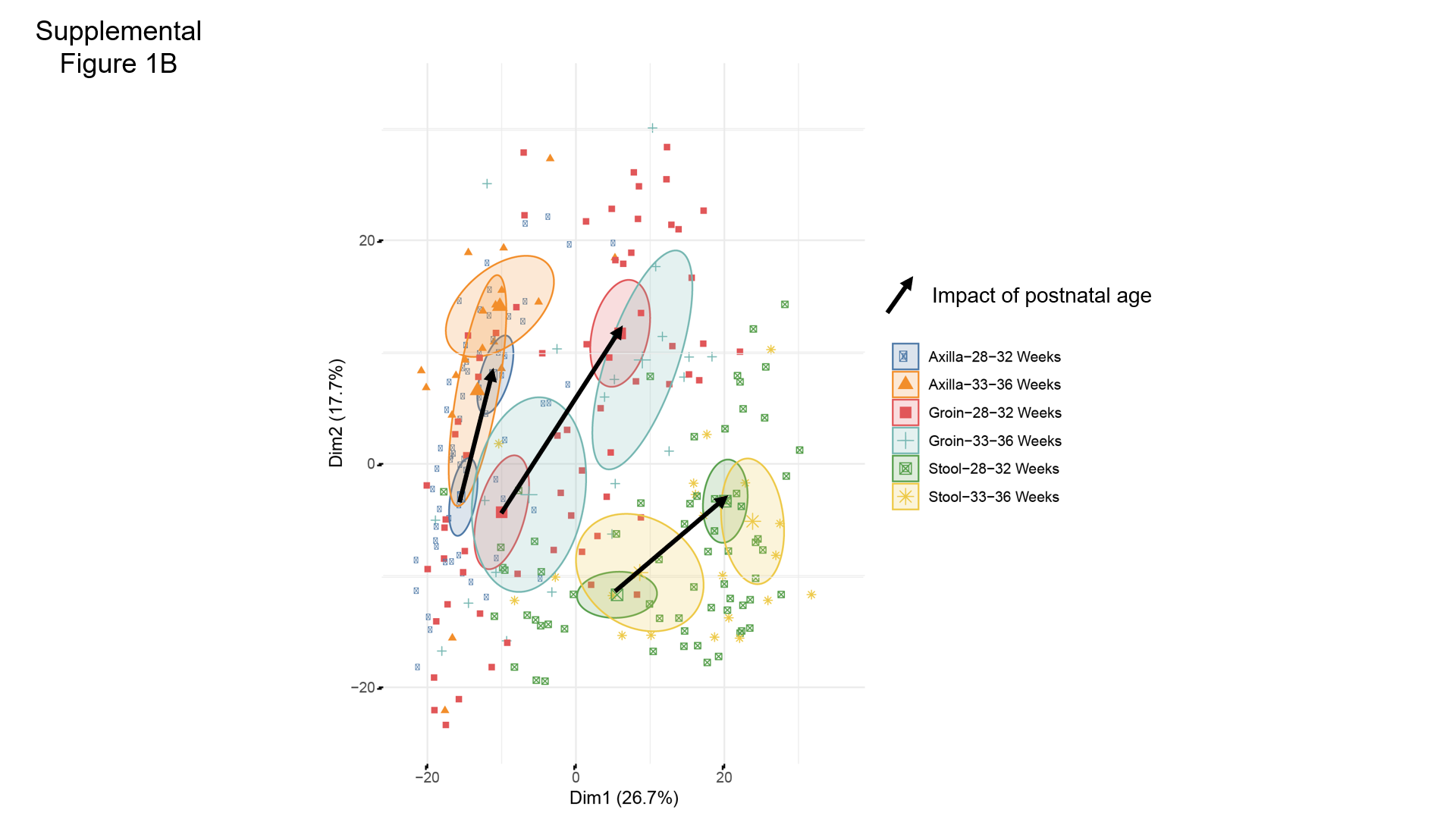


Supplemental Figure 1B. Impact of postnatal age on microbiome structure. Postnatal age had a significant impact on microbiome composition at all body sites among gestation cohorts (*P* <0.001).


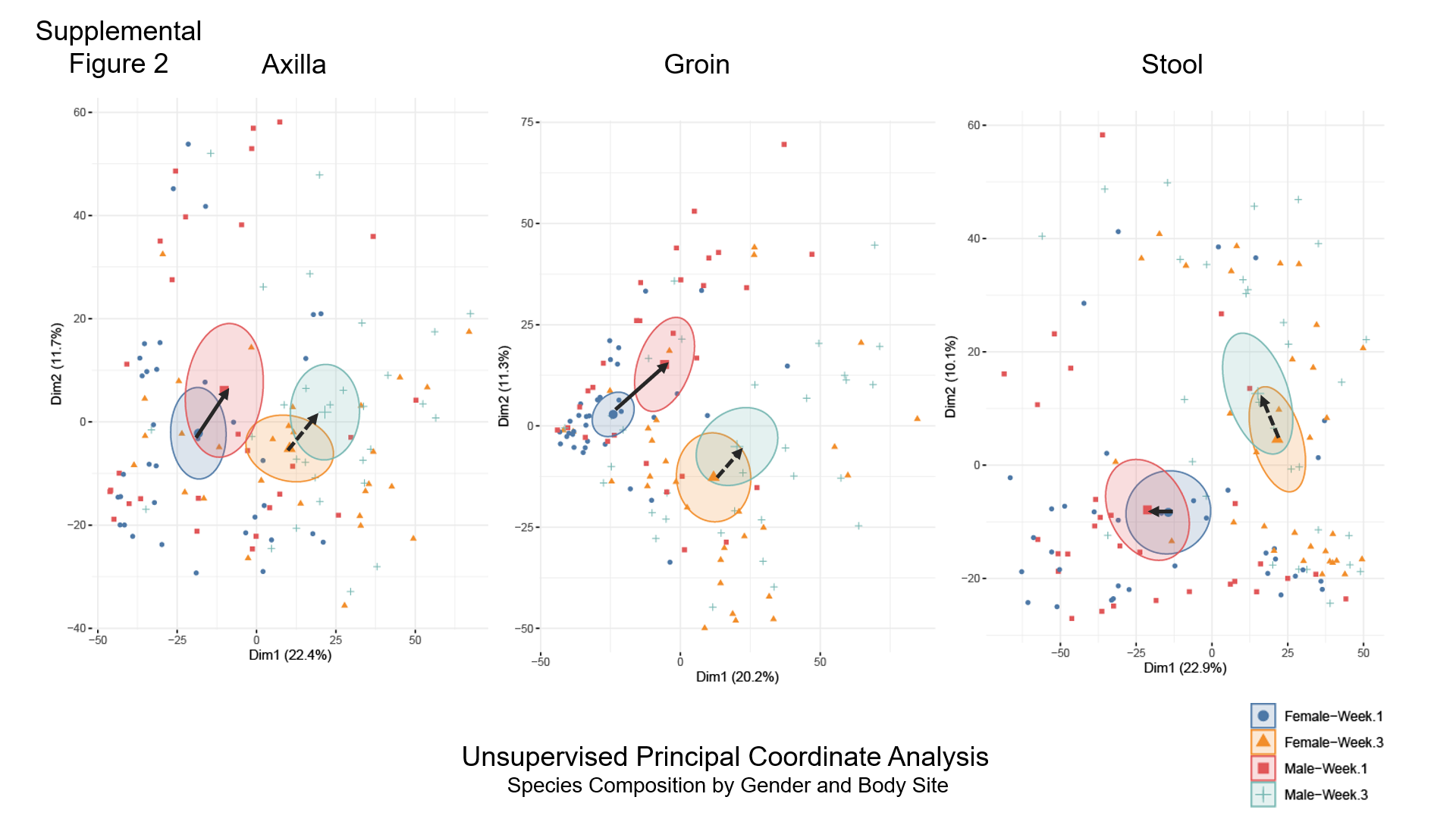


Supplementary Figure 2. The contribution of gender to microbiome composition at all body sites and found the only time point with difference in gender was groin at Week 1 (p=0.002, other p>0.05).


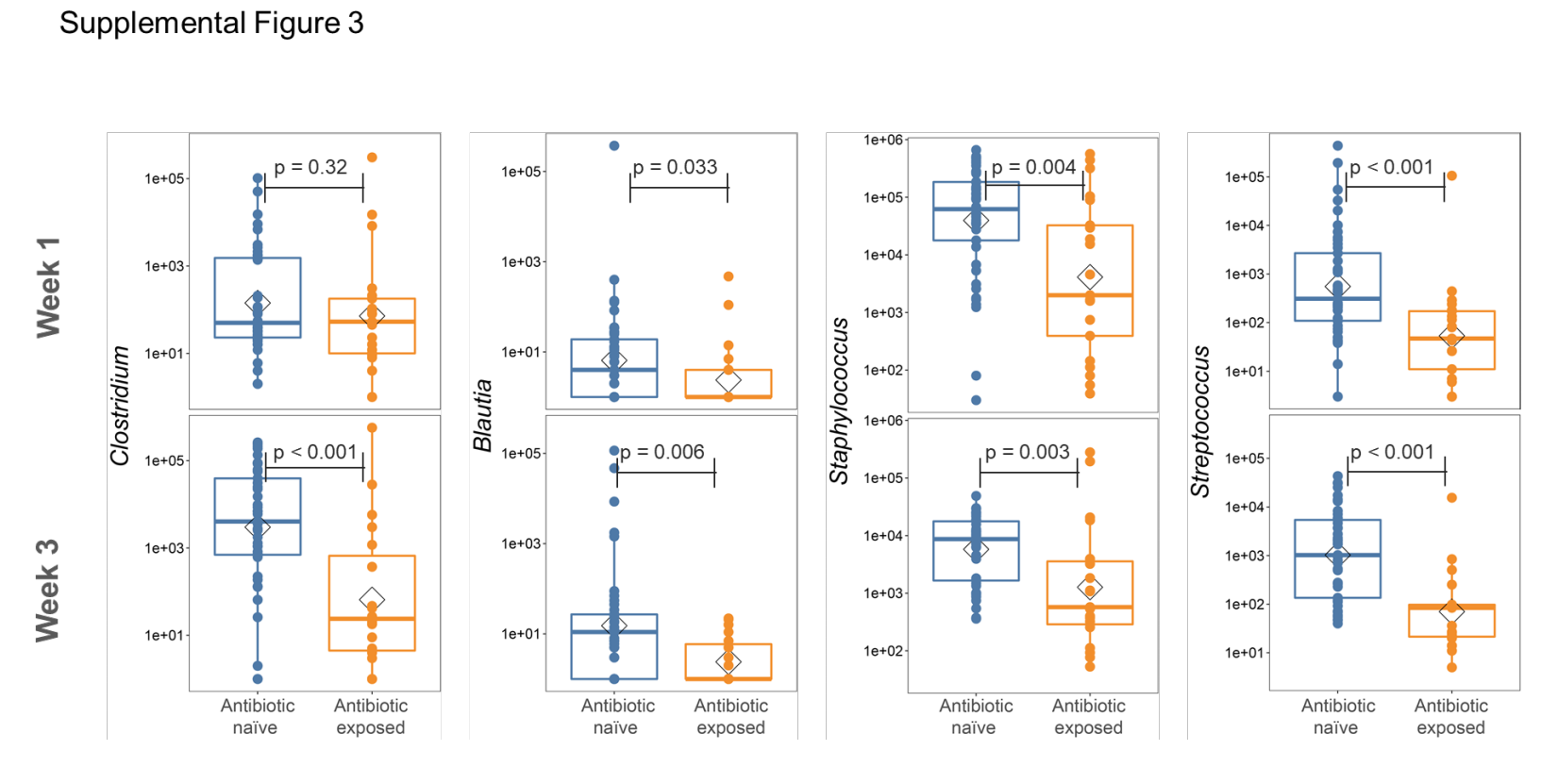


Supplemental Figure 3. Antibiotic exposure was associated with altered abundance of several genera. ZINB-GLMM was used to identify genera that were statistically significantly different in the preterm infant gut microbiome in antibiotic-naïve and -exposed infants at weeks 1 and 3. After accounting for gestational age, maternal antibiotics, breast milk receipt, and delivery mode several genera were significantly associated with antibiotic receipt. Demonstrative genera include Clostridium, Blautia, Staphylococcus and Streptococcus. p-values were calculated by Wilcoxon-ranked sum test.


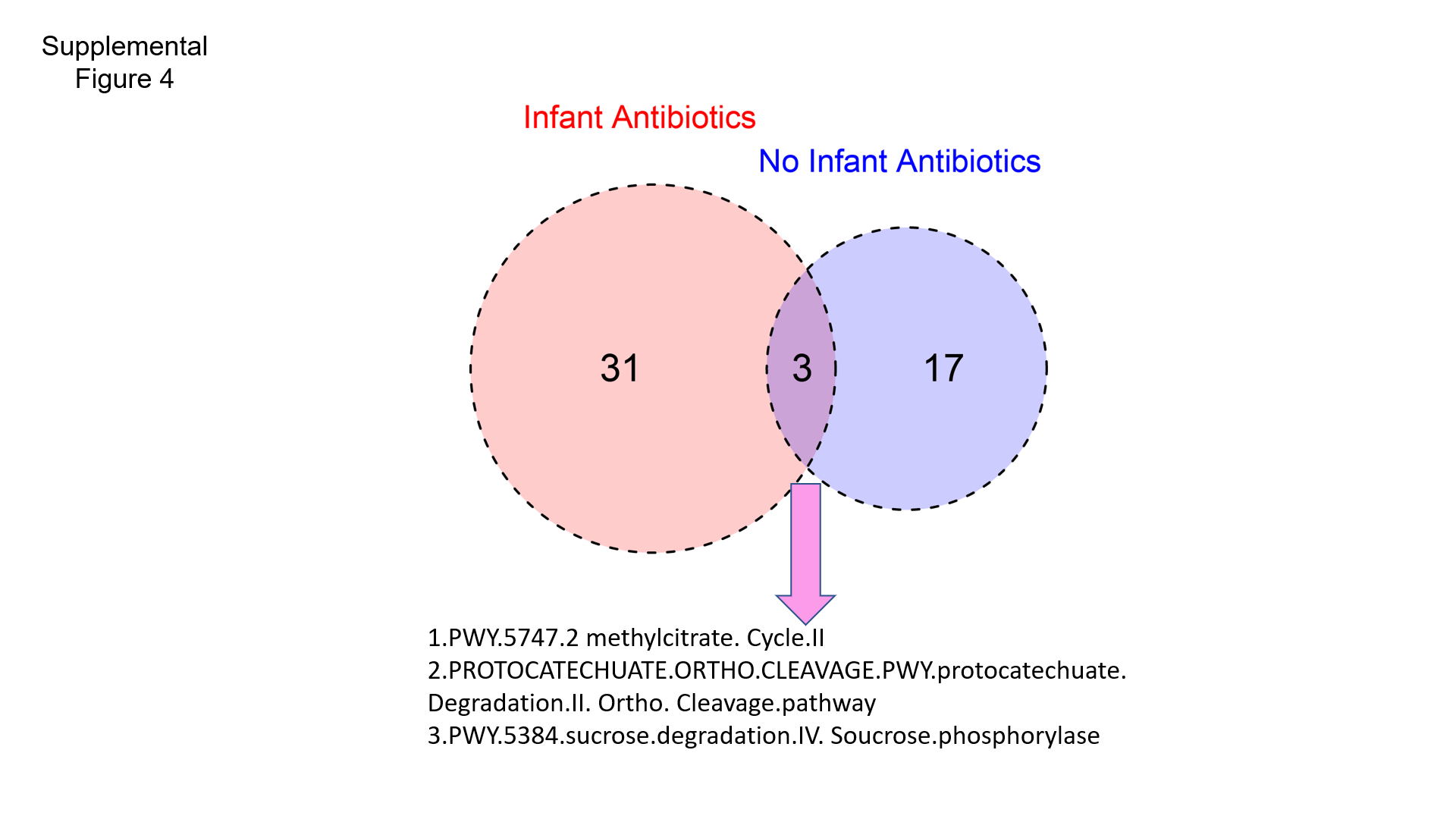


Supplemental Figure 4. Microbial metabolic pathway abundance and enrichment from Week 1 to Week 3 were compared between antibiotic-exposed and antibiotic-naïve preterm infants using MRPP and GLMM, as described in the text. There was minimal overlap in the metabolic pathways enriched from Week 1 to Week 3 in antibiotic-naïve and antibiotic-exposed infants.
